# 3D Fluid-Structure Interaction Simulation of Aortic Valves Using a Unified Continuum ALE FEM Model

**DOI:** 10.3389/fphys.2018.00363

**Published:** 2018-04-16

**Authors:** Jeannette H. Spühler, Johan Jansson, Niclas Jansson, Johan Hoffman

**Affiliations:** Department of Computational Science and Technology, School of Computer Science and Communication, KTH Royal Institute of Technology, Stockholm, Sweden

**Keywords:** fluid-structure interaction, finite element method, Arbitrary Lagrangian-Eulerian method, parallel algorithm, blood flow, patient specific heart model

## Abstract

Due to advances in medical imaging, computational fluid dynamics algorithms and high performance computing, computer simulation is developing into an important tool for understanding the relationship between cardiovascular diseases and intraventricular blood flow. The field of cardiac flow simulation is challenging and highly interdisciplinary. We apply a computational framework for automated solutions of partial differential equations using Finite Element Methods where any mathematical description directly can be translated to code. This allows us to develop a cardiac model where specific properties of the heart such as fluid-structure interaction of the aortic valve can be added in a modular way without extensive efforts. In previous work, we simulated the blood flow in the left ventricle of the heart. In this paper, we extend this model by placing prototypes of both a native and a mechanical aortic valve in the outflow region of the left ventricle. Numerical simulation of the blood flow in the vicinity of the valve offers the possibility to improve the treatment of aortic valve diseases as aortic stenosis (narrowing of the valve opening) or regurgitation (leaking) and to optimize the design of prosthetic heart valves in a controlled and specific way. The fluid-structure interaction and contact problem are formulated in a unified continuum model using the conservation laws for mass and momentum and a phase function. The discretization is based on an Arbitrary Lagrangian-Eulerian space-time finite element method with streamline diffusion stabilization, and it is implemented in the open source software Unicorn which shows near optimal scaling up to thousands of cores. Computational results are presented to demonstrate the capability of our framework.

## 1. Introduction

The World Health Organization (WHO, 2014) has identified cardiovascular disease as the major cause for death in the world. Therefore, developing new ways to support early diagnosis of cardiac dysfunction is of vital importance. *In vivo* and *in vitro* studies offer valuable information on the relationship between the blood flow (hemodynamics) and cardiac disease, and advances in computational fluid dynamics (CFD) and high performance computing (HPC) enable the usage of computer simulation as an important tool to further enhance our understanding of this relationship.

The field of cardiac modeling is extensive, and highly interdisciplinary. It is therefore important to be clear on what the research is aiming for. Our goal is to develop a framework for simulating the intraventricular blood flow, where specific properties such as fluid-structure interaction (FSI) of the aortic valve can be implemented in a modular way without extensive efforts. In Spühler et al. (2015) we focus on the aspect of fluid mechanics, and present a computational model of the blood flow in the left ventricle (LV) of the heart. The movement of the wall is based on ultrasound measurements and an Arbitrary Lagrangian-Eulerian (ALE) space-time finite element method is used to simulate the blood flow by solving the incompressible Navier-Stokes equations. The opening and closing of the mitral and aortic valves are modeled by time-dependent velocity and pressure boundary conditions. In this paper, we present an extension of this work by embedding different geometrical models of aortic valves in the LV and the aorta. Prototypes of a biological valve and bileaflet mechanical heart valve (BMHV) are modeled. While surgical treatments of valvular diseases are firmly established, many decisive factors for the performance of the implant are not fully understood yet. Numerical simulations provide an important insight to the interaction between the blood flow and the leaflets which can be applied to optimize the design of BMVHs or improve technologies as transcatheter aortic valve replacement (Wu et al., 2016). The fluid-structure interaction problem is described by a unified continuum model, using the conservation laws for mass and momentum and a phase function, which is a novel approach for simulating valve motions. The Navier-Stokes equations are solved by an ALE space-time finite element method with streamline diffusion stabilization implemented in Unicorn (Hoffman et al., 2012), which is part of the open source software framework FEniCS-HPC (Jansson, 2013).

This paper is structured as follows. In section 2 we describe the different components and functions of an anatomical aortic valve. section 3 explains the mathematical equations and the numerical method. In section 4, we specify the mechanical and biological aortic valve model we use in our simulations. The numerical results are presented in section 5 and we conclude our paper in section 6 by summarizing our findings and discuss possible steps of future work.

## 2. Modeling the aortic valve

The left ventricle possesses a mitral and an aortic valve, each of them consisting of two and three leaflets respectively. The valves ensure unidirectional flow and prevent the blood to flow back. The opening and closing of the valves are mainly controlled by the pressure gradient between the ventricle and the adjacent chamber. One edge of the biological leaflet is completely attached to the inner wall of the heart. The free edge of the mitral valve is connected to the papillary muscles by the chordae tendineae. The aortic leaflets do not have such fibrous tissue connections and they open and close passively due to the flow.

**Figure 1:**
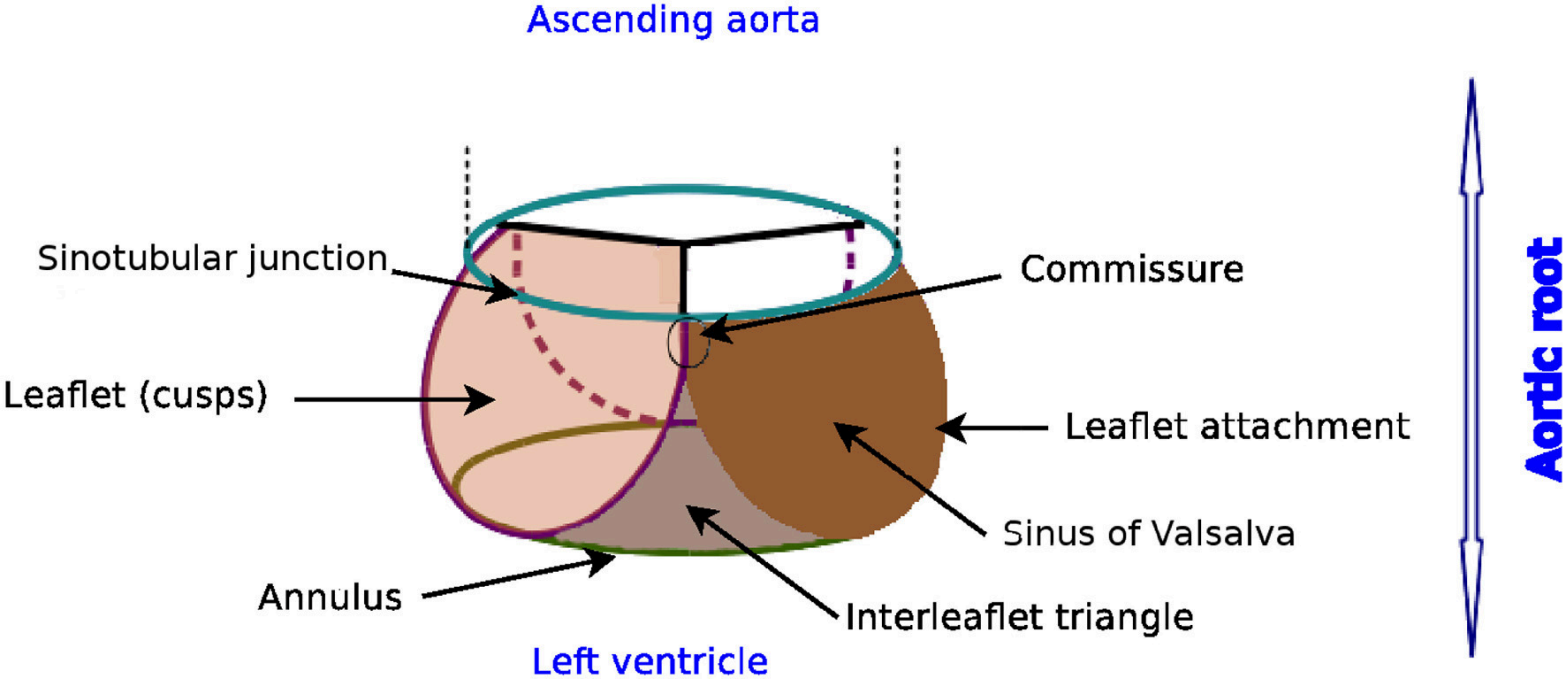
Glossary of the aortic root, author's own drawing based on Sievers et al. (2012). Annulus, leaflets, leaflet attachment, sinotubular junction, interleaflet triangle and sinus of Valsalva are the different components of the aortic root. The aortic valve consists of the three leaflets only.

The nomenclature of the different components of the aortic root can vary remarkably as revealed by Sievers et al. (2012). We apply the definitions proposed in Sievers et al. (2012), as indicated in Figure 1. The aortic root is situated between the left ventricle and the ascending aorta, and is bordered by the annulus and the sinotubular junction. The three bulges just above the annulus are referred as sinus of Valsalva. The aortic valve contains three leaflets which are attached to the aorta wall. The point of contact where two leaflets meet at the root wall is called commissure and the surface of contact at the free edge is known as coaptation.

## 3. Mathematical model and numerical method

In order to put our approach in context, we review different models for simulating the FSI of the blood flow around the aortic valve. Usually, the structure model is formulated in the Lagrangian coordinate system whereas fluid flow is described in the Eulerian coordinate system. At the common interface of the two models, the following kinematic and dynamic constraints have to be satisfied by the velocity **u** and the stress **τ**:

(1) $$u_{f}=u_{s}\text{ (kinematic constraint, continuity of the velocity)},$$

(2) $$\tau_{s}\cdot n=\tau_{f}\cdot n\text{ (dynamic constraint, continuity of the normal stresses)}.$$

The subscript indicates whether the variable is defined in the solid (*s*) or in the fluid (*f*) part respectively, while **n** is a unit vector normal to the interface. We denote vectors and matrices with bold letters. FSI simulations can roughly be categorized as *moving or fixed mesh methods* and *partitioned or monolithic approach* as presented in Borazjani et al. (2008).

### 3.1. Discretization of the coupled problem

For *fixed mesh methods*, the fluid and structure domains are discretized in a non-boundary conforming matter. Since the structure is spatially disconnected from the fixed background mesh, it is crucial to efficiently trace and move the interface between the solid and the fluid domain. The interface can be discretized with a set of markers and tracked by a Lagrangian method (*front tracking*) or represented by contours or level sets of a scalar function (*front capturing*). Fixed mesh methods were pioneered by Peskin and McQueen (Peskin, 1972; McQueen and Peskin, 2000) introducing the concept of *immersed boundary methods*, where body forces are imposed on the fluid domain to account for the interaction between the fluid and the structure. Large structural deformations are manageable, but the solution at the interface can be diffuse. This disadvantage can be lessened by e.g., increasing the mesh resolution in the vicinity of the immersed boundary as done by Griffith (2012), or by treating the boundary as a sharp interface as in e.g., Borazjani et al. (2008); Udaykumar et al. (1999); Mittal and Iaccarino (2005); Gilmanov and Sotiropoulos (2005), and Xia et al. (2009). *Fictitious domain methods* is a another class of fixed mesh methods where Lagrange multipliers account for the kinematics constraints between the fluid and solid domain, see e.g., Glowinski et al. (1999); van Loon et al. (2005); De Hart et al. (2003), and Astorino et al. (2009).

In *moving mesh methods*, the computational mesh conforms to the deformation of the solid domain, and is typically represented by an Arbitrary Lagrangian Eulerian formulation. The strength of the moving grid methods can be found in its accuracy and clearly defined coupling condition, as the mesh is aligned with the fluid-structure interface. A good smoothing algorithm or local remeshing is needed to keep the quality of the computational mesh.

Reviewing the literature of aortic valve simulations, we came across the following work which apply an ALE approach: Bolger et al. (2007) and Penrose and Staples (2002) simulate the flow past a geometrically reduced mechanical valve prosthesis taking advantage of its symmetrical form; in Dumont et al. (2007) two commercially available bileaflet mechanical heart valves are compared regarding hemodynamics and thrombogenic performance; Guivier-Curien et al. (2009) employs particle image velocity measurements to quantitatively and qualitatively compare experiments and numerical simulations; the FSI model of Choi and Kim (2009) provides detailed flow information and leaflet behavior of a BMHV; Morsi et al. (2007) analyzes the fluid dynamics of a trileaflet heart valve but only for the initial opening phase.

### 3.2. Coupling strategies

Depending on whether the structure and fluid problems are solved simultaneously or separately, the FSI solver can be classified as *monolithic* or *partitioned*. The FSI approach is called *monolithic* if the fluid and solid problems are solved as one single system where no matching of the data is required at the interface.

In a *partitioned* approach, there are two different solvers simulating the fluid and the solid part respectively. If the coupling between the solvers is explicit in time then the coupling is loose. The loose coupling has low computational cost, but the simulation may become unstable. To overcome these instability issues, the partitioned problem can be formulated implicit in time, introducing an iteration loop at each time step until a dynamic equilibrium between the fluid and solid is achieved. Data exchange between the fluid and solid part in this implicit algorithm is called a strong coupling.

### 3.3. Unified continuum model

We now specify our ansatz, which corresponds to a monolithic, moving mesh method. An elaborate description can be found in Jansson et al. (2011) at full length. Here, we only describe the main features.

Where the size of the vessel is much larger than the size of a red blood cell, the blood flow can be modeled as an incompressible Newtonian fluid (Quarteroni et al., 2014). The governing equations are the Navier-Stokes equations. The dynamic viscosity is chosen as μ = 0.0027*Pa* · *s* and the blood density ρ = 1, 060*kg*/*m*^3^ (Di Martino et al., 2001). In small domains, as the region around the revolute joints of a mechanical heart valve, non-Newtonian effects might have to be incorporated in the model, but these flow features are not targeted in this work.

With the aim of establishing a framework that allows for general formulation and implementation of different models, while applying adaptive error control for realistic 3D applications, a so-called unified continuum model for FSI was developed. The model is described by the conservation laws of mass and momentum for an incompressible continuum, where a stress and phase variable define the properties of the continuum.

Let Ω^*t*^ ⊂ ℝ^3^ be a time-dependent domain with $t\in I:=\left[0,\hat{t}\right]$. Our goal is to determine **u**(**x**, *t*) : Ω^*t*^ → ℝ^3^, where Ω^*t*^ encompasses both the solid and the fluid domain and **u** defines the fluid velocity in the fluid part and the deformation velocity in the structure part:

(3a) $$\rho (\dot{u}+((u-m)\cdot \nabla )u)=\nabla \cdot \tau (u,p)\;(x,t)\in \Omega^{t}\times I,$$

(3b) $$\nabla \cdot u=0\;(x,t)\in \Omega^{t}\times I.$$

Here **τ** is the stress tensor and **m** identifies the mesh velocity in the ALE formulation. In the solid, we choose **m** to be the material velocity of the structure. In the remaining part of the mesh, **m** is determined by the mesh smoothing algorithm applied to uphold the quality of the mesh.

The constitutive laws are defined via the stress term, where the phase function θ is set to zero in the solid domain and to one in the fluid domain:

(4) $$\tau =\tau_{D}-pI,$$

(5) $$\tau_{D}=\theta \tau_{f}+(1-\theta )\tau_{s},$$

(6) $$\tau_{f}=2\mu_{f}\epsilon (u),$$

(7) $$D_{t}\tau_{s}=2\mu_{s}\epsilon (u)+\nabla u\tau_{s}+\tau_{s}\nabla u^{T},$$

(8) $$\epsilon (u)=\frac{1}{2}(\nabla u+\nabla u^{T}).$$

The kinematic constraint **u_f_** = **u_s_** is satisfied implicitly by the continuity of the velocity field **u** for the unified continuum. The dynamic constraint is weakly enforced by applying integration by parts on the stress term and setting it to zero in the weak formulation.

This approach allows us to use the same discretization method, stabilization technique and mesh deformation algorithm as for a pure fluid problem.

### 3.4. Time and space discretization

Let $0:=t^{0}<t^{1}<\cdots <t^{N}:=\hat{t}$ be a sequence of discrete time steps, with associated time intervals *I*^*n*^: = (*t*^*n*−1^, *t*^*n*^] of length *k*^*n*^: = *t*^*n*^ − *t*^*n*−1^.

We introduce the space-time slab *S*^*n*^: = Ω^*t^n^*^ × *I*^*n*^, and let *T*^*n*^ = {*K*} denote the spatial discretization of Ω^*t^n^*^. **U**^*n*^ is the discrete velocity, *P*^*n*^ is the discrete pressure, and *h*^*n*^ specifies the maximal diameter of the cells *K*∈*T*^*n*^.

We choose the finite element function space of piecewise linear functions *W*^*n*^ ⊂ *H*^1^(Ω^*t^n^*^), where

(9) $$H^{1}(\Omega^{t^{n}}):\text{​}=\{v\in L^{2}(\Omega^{t^{n}})|\frac{\partial v}{\partial x_{k}}\in L^{2}(\Omega^{t^{n}}),k=1,2,3\},$$

(10) $$W^{n}:\text{​}=\{v\in C(\Omega^{t^{n}})|v\in P^{1}(K),\forall K\in T^{n}\},$$

(11) $$W_{0}^{n}:\text{​}=\{v\in W^{n}|v=0\text{ on }\partial \Omega^{t^{n}}\},$$

(12) $$W_{0}^{n}:\text{​}={\left[W_{0}^{n}\right]}^{3}.$$

We identify the discrete solution for velocity and pressure as $\hat{\text{U}}=\left(\text{U},P\right)$, the discrete stress for both the fluid and the solid as T, the discrete mesh velocity as **M**, and the test function as $\hat{\text{v}}=\left(\text{v},q\right)$. In time, we choose **U** to be piecewise linear, and *P*, **v** and *q* to be piecewise constant.

Based on these definitions and assuming homogeneous Dirichlet boundary condition for the velocity, the spatially and temporally discretized variational formulation of Equation (3) reads as follows: for each space-time slab *S*^*n*^, find (**U^n^**, *P*^*n*^): = (**U**(*t*^*n*^), *P*(**t**^**n**^)) with ${\text{U}}^{n}\in {\text{W}}_{0}^{n}$ and *P*^*n*^ ∈ *W*^*n*^, such that:

(13) $$\begin{array}{l} \left(\rho k_{n}^{-1}(U^{n}-U^{n-1})+(\rho ({\bar{U}}^{n}-M^{n})\cdot \nabla ){\bar{U}}^{n},v)+(T^{n}:\nabla v\right)\\ \;+SD_{\delta }({\bar{U}}^{n},M^{n},P^{n},v,q,\rho )=0, \end{array}$$

for $\forall \left(\text{v},q\right)\in {\text{W}}_{0}^{n}\times W^{n}$, where ${\bar{\text{U}}}^{\text{n}}=\frac{1}{2}\left({\text{U}}^{n}+{\text{U}}^{n-1}\right)$ and (.,.) denotes the *L*^2^(*S*^*n*^)-inner product.

To stabilize the convection dominated problem (3), we use a simplified Galerkin/least-square method, where we drop the time derivative and the diffusion term, and we define *SD*_δ_ as

(14) $$\begin{array}{l} SD_{\delta }({\bar{U}}^{n},P^{n},v,q,\rho )=\\ \left(\delta_{1}\rho ((({\bar{U}}^{n}-M^{n})\cdot \nabla ){\bar{U}}^{n}+\nabla P^{n}),\rho (({\bar{U}}^{n}-M^{n})\cdot \nabla )v+\nabla q\right)\\ +(\delta_{2}\nabla \cdot {\bar{U}}^{n},\nabla \cdot v). \end{array}$$

The stabilization parameters are chosen as $\delta_{2}=\kappa_{2}\rho h^{n}|{\text{U}}^{n-1}|$ and $\delta_{1}=\kappa_{1}\rho^{-1}{\left(k_{n}^{-2}+|U^{n-1}-M^{n-1}{|}^{2}h_{n}^{-2}\right)}^{-1/2}$, where κ_1_, κ_2_ are problem independent positive constants of order *O*(1). By applying the midpoint quadrature rule in time, we obtain a Crank-Nicolson time-stepping scheme. We use Bi-CGStab with a block Jacobi preconditioner where each sub-block is solved with ILU(0).

### 3.5. Smoothing algorithms

Due to the fluid-structure interaction of the aortic valve and the pumping blood flow from the left ventricle, it is crucial for an ALE-method to have a suitable method to adjust an existing mesh. There are different ways to enhance and optimize the quality of the mesh, which may involve e.g., swapping faces and edges, or changing the number of vertices.

Meshing algorithms, which involve change of topology or the number of mesh cells, are not suitable for time-dependent, parallel computing. Therefore, it is preferable to use a mesh adaptivity method which omits the necessity or at least minimizes the frequency of remeshing.

To keep a good mesh quality, while limiting the computational cost, our solver combines a linear and a nonlinear mesh smoothing algorithm. The linear smoother accounts for the rough overall re-distribution of the vertices, while the nonlinear smoother optimizes locally the mesh based on the quality of the cells.

#### 3.5.1. Linear smoother

The linear smoother solves a linear elastic equation in the fluid domain for the mesh velocity, which corresponds to a Poisson equation with Dirichlet boundary conditions given by the structure velocity on the fluid-structure interface, where the vertices are diffusively relocated over the domain. Although it is a simple and fast method, there is no guarantee that improvement is achieved since the equation does not take into consideration the quality of the cells in the mesh.

#### 3.5.2. Nonlinear smoother

To locally enhance distorted cells, we describe the deformation of the mesh using a nonlinear elasticity problem, and weight the stiffness of the model by a quality measure *Q*(*K*) of each cell *K* in the mesh *T*^*n*^:

(15) $$Q(K):\text{​}=\frac{||F|{|}_{F}^{2}}{\det{\left(F\right)}^{2/d}d},$$

where *d* specifies the dimension of the spatial domain and ||.||_*F*_ the Frobenius norm. *F* denotes the deformation gradient between *K*∈*T*^*n*^ and a scaled equilateral reference cell.

By weighting the equation by *Q*(*K*) and advancing the partial differential equation toward its equilibrium, the mesh is improved toward its goal of optimal shape. A more detailed description is elaborated in Jansson et al. (2011).

To limit the computational cost, the nonlinear smoother is stopped after a certain number of “pseudo” time steps $\tilde{k}$ before a stationary solution is obtained. Depending on the quality of the mesh *T*^*n*^, the total number of pseudo time steps can be adapted to achieve a desired quality.

### 3.6. Modeling of contact

In order to simulate the closing of a heart valve, an algorithm needs to be implemented to both detect collision and to simulate contact. Our approach is derived from the idea to describe the fluid-structure interaction as a unified continuum. We model contact implicitly by switching fluid cells to solid cells as soon as contact is detected. Collision is detected by solving an Eikonal equation for the distance between two solid surfaces.

**Figure 2:**
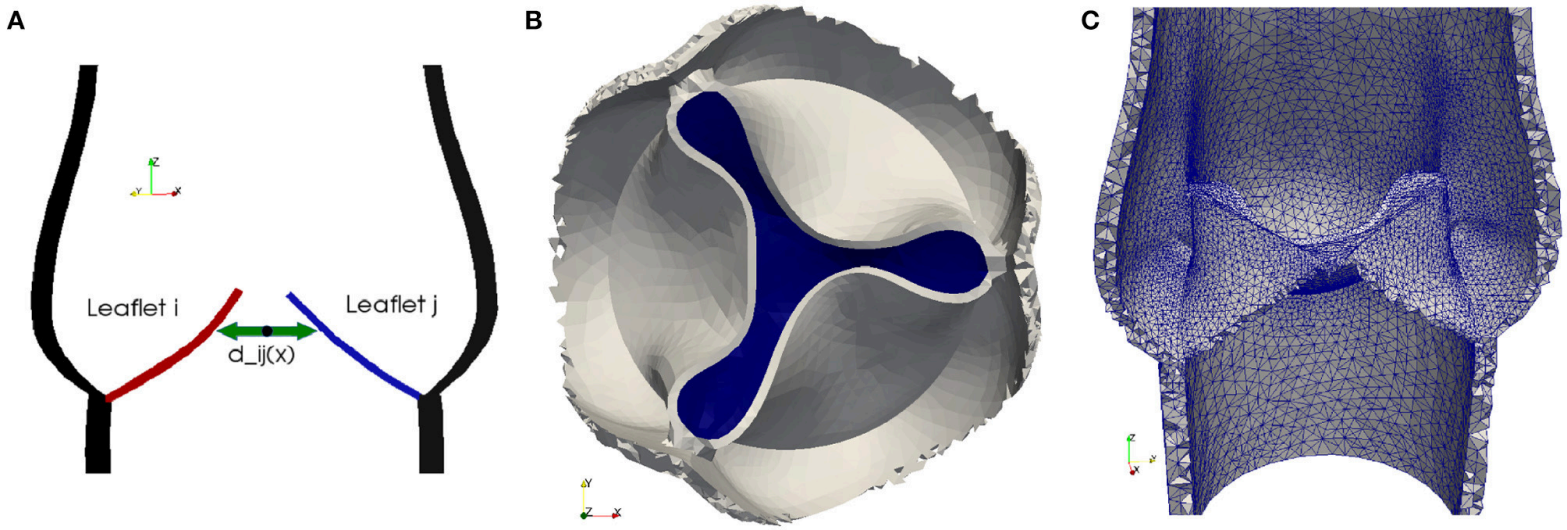
To detect collision we calculate the distance *d*_*ij*_ between the leaflets *L*^*j*^ and *L*^*i*^ for *i, j* = 1, 2, 3 **(A)**. As soon as the minimal distance is below a certain threshold, the valve opening is closed. A 2D-surface (blue) is included to model a proper closure **(B)** and the geometric model of the valve opening is closed by marking the cells directly attached to the 2D-surface as solid **(C)**.

In order to detect contact between two leaflets of a native valve, we calculate the minimal distance $d_{min}:=min_{ij,i\neq j}\left\{d^{ij}\right\}$ between the leaflets *L*^*j*^ and *L*^*i*^ for *i, j* = 1, 2, 3, as illustrated in Figure 2A. To model a proper closure of the leaflets, we include a 2D-surface in our volume mesh, which covers the entire valve opening, and as soon *d*_*min*_ is below a certain threshold, all cells directly attached under the 2D-surface are marked as solid, as shown in Figures 2B,C. Since the closing moment of a healthy valve is very short, we argue that it is acceptable to cover the whole opening at once. The contact is released at the beginning of the subsequent contraction phase (systole) of the left ventricle.

### 3.7. Computational tools

Nowadays high performance computing is an essential part of computational science. The Heart-FSI solver is implemented in the HPC branch (Jansson, 2013) of the open source FEM library DOLFIN (Logg and Wells, 2010) and the adaptive flow solver Unicorn (Hoffman et al., 2012). Both libraries have successfully been used to efficiently solve large scale industrial problems as described in e.g., Jansson et al. (2011) and Vilela de Abreu et al. (2016).

The simulations were performed on Beskow, a Cray XC40 system, where each node has two CPUs (Intel E5-2698v3) with 16 cores. All volume meshes are created in ANSA (2014), a computer-aided engineering tool for pre-processing.

## 4. Valve models

In the subsequent paragraphs, we describe how we model native and bileaflet mechanical heart valves (BMHV) embedded in the left ventricle and ascending aorta. For each case, we detail the geometry and specify the material as well as the initial and boundary conditions.

### 4.1. Native valve

#### 4.1.1. Geometry

The geometry of the aortic root has been studied, where geometrical parameters are optimized to resemble the function of a trileaflet valve (Swanson and Clark, 1974). Our model is based on such an optimized geometry proposed by Thubrikar (1990).

**Figure 3:**
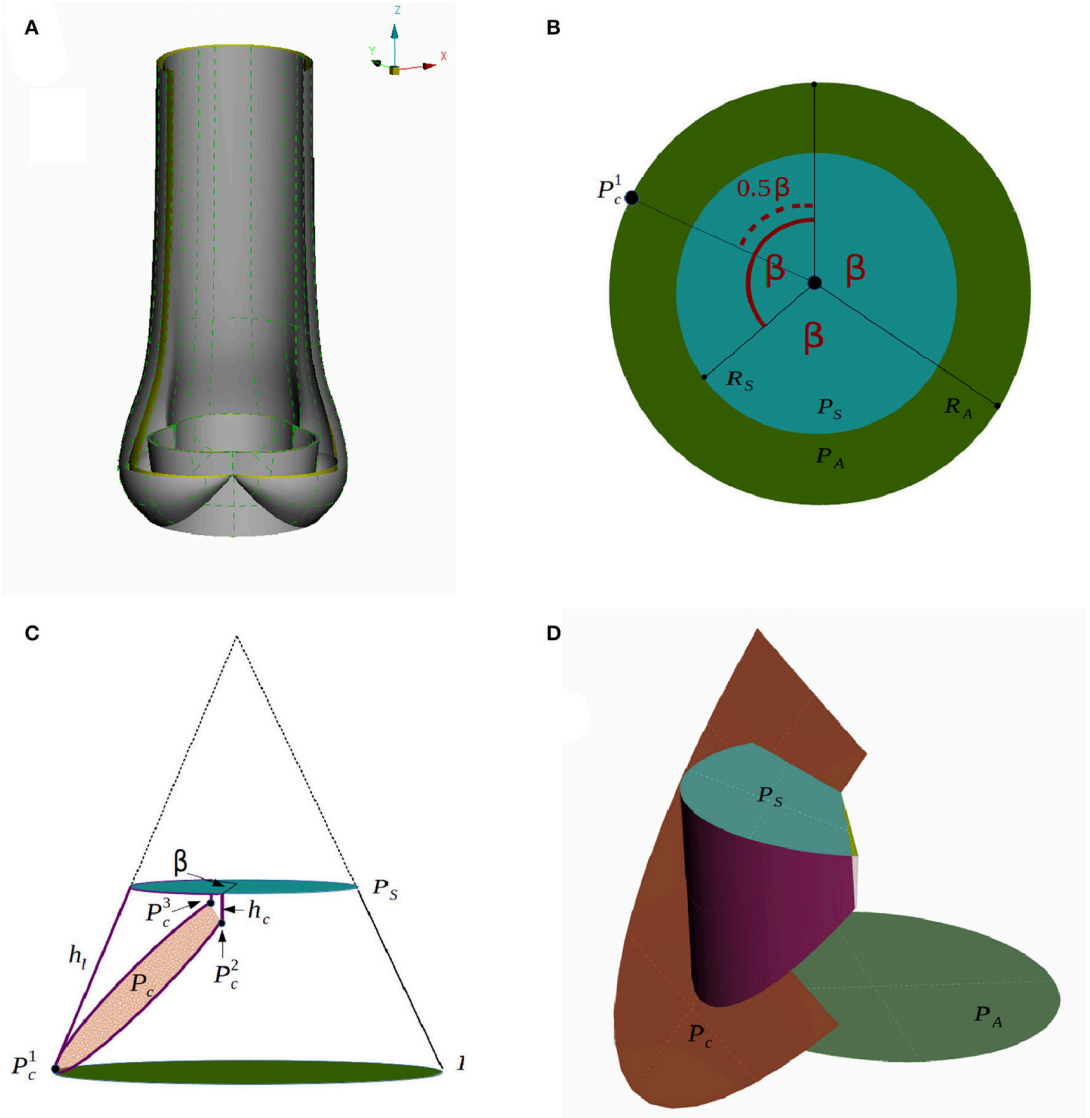
A CAD model of an idealized native aortic root **(A)** and its parameters in top down view **(B)** and side view **(C)** are depicted. The creation of the surface of one leaflet is illustrated in **(D)**.

We generate a computer-aided design (CAD) model of an idealized native aortic root based on a small set of parameters which can be personalized to a particular patient. The aortic root generator is a set of Python scripts for Rhinoceros 5 (Rhinoceros, 2016) that outputs an aortic root in a fully open valve configuration, as presented in Figure 3A. The model parameters are illustrated in Figures 3B–D.

We assume that the aortic root has a threefold symmetry around the z-axis and label the rotational angle by β as depicted in Figure 3B. The plane *P*_*A*_ at the annulus and the plane at the sinotubular junction *P*_*S*_ are assumed to be parallel. The inner radius at the annulus *R*_*A*_, the inner radius at the sinotubular junction *R*_*S*_ and the length of the leaflet in the open position *h*_*l*_, are used to define a truncated cone as shown in Figure 3C. To find the leaflet attachment and the leaflet surface the cone is cut by the plane *P*_*c*_, which is defined by three points $P_{c}^{1},P_{c}^{2}$, and $P_{c}^{3}$, see Figures 3C,D. These points are determined by the height of the commissure *h*_*c*_ and the opening angle β. We attach a cylinder with radius *R*_*S*_ to the aortic root to model the beginning of the ascending aorta. Geometrical parameters for the sinus of Valsalva are not considered as modifiable yet. The thickness is acquired by copying, scaling and translating surfaces. The parameter values used in the simulations are listed in Table 1.

**Table 1:** Model parameters used for generating the native aortic root geometry.

**Model parameters**	**Parameter**	**Value [mm]**
Inner base radius at the annulus	*R*_*A*_	20
Inner radius at the sinotubular junction	*R*_*S*_	22
Leaflet height	*h*_*l*_	20
Height of commissure	*h*_*c*_	6
Thickness of leaflet	*t*_*l*_	1

#### 4.1.2. Material

The leaflets are made of a very thin, flexible and inextensible material. The fibers in an aortic leaflet are aligned in the circumferential direction (Swanson and Clark, 1974), and the mechanical properties vary in different parts of the aortic valve (Kasyanov et al., 1985). In the framework of this work, it is sufficient to assume the solid material to be homogeneous. As material model we choose an incompressible, neo-Hookean material. At this point of development, the material parameters are set to $\mu_{s}=3.3\cdot 10^{3}MPa$ and ρ = 1, 000*kg*/*m*^3^. Although these parameters do not conform with realistic values yet, typical characteristics of the flow and valve dynamics can be captured.

#### 4.1.3. Initial and boundary conditions

**Figure 4:**
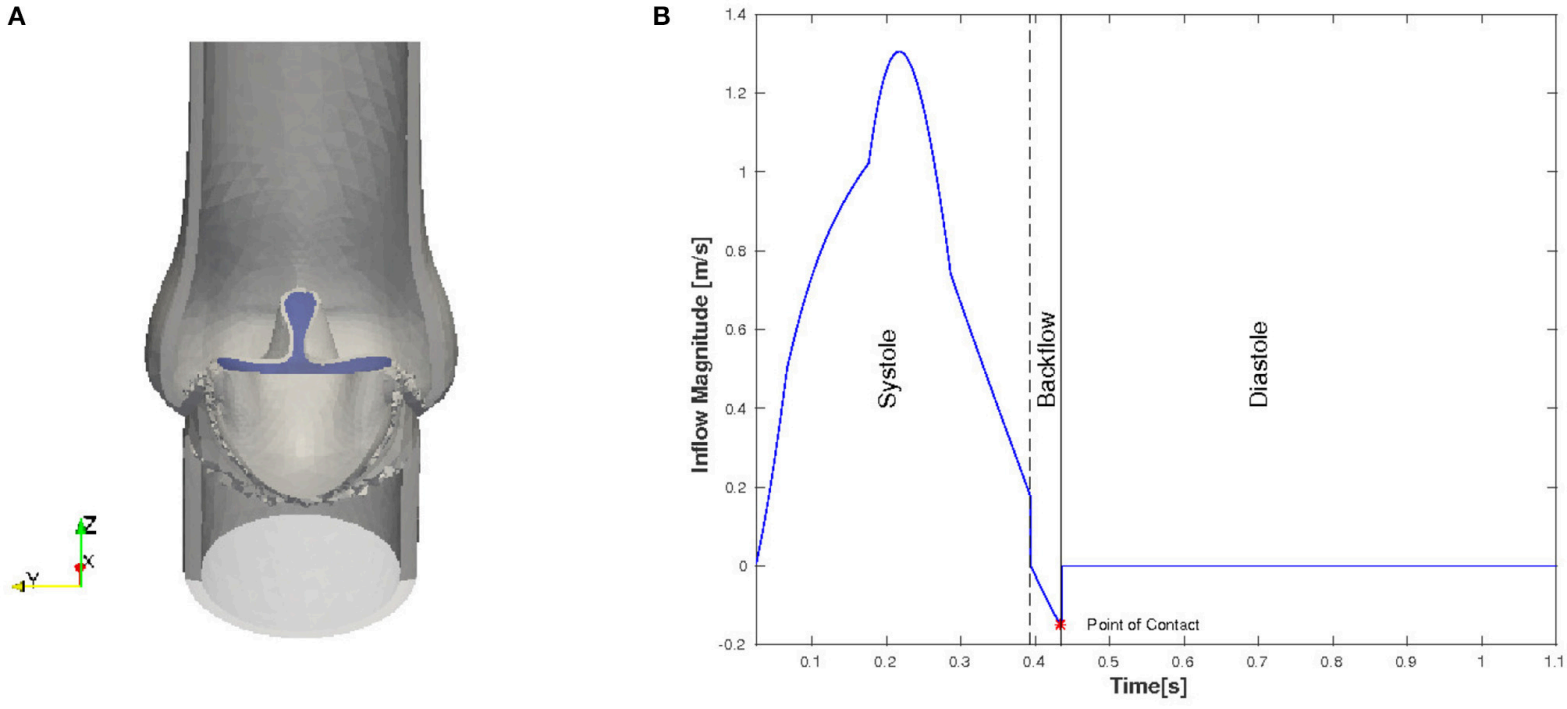
Initial and boundary conditions for the native valve: starting configuration for the simulation **(A)** and the magnitude of the inflow plotted against time **(B)**.

Even though in the initial geometry the valve is in a fully open position, the leaflets are pushed into a starting configuration to facilitate the movement of the leaflets. In order to remove excessive leaflet material resulting from the deformation, we prescribe a constant, initial stress in radial direction such that the material behaves like a contracting balloon which was stretched. The starting position for our simulations with initial radial stress 4 Pa is shown in Figure 4A. The stress is reset for the FSI simulation.

We only consider the two major phases, systole and diastole, and one heart cycle lasts for 1.124 s. The inflow profile is flat and the magnitude is adopted from the left ventricle flow simulations presented in Spühler et al. (2015). At the end of systole, the direction of the inflow is inverted to create a backflow which is physiologically consistent and helps the valve to close. The time-dependent inflow magnitude is plotted in Figure 4B. Diastole starts when the valve is closed and the inflow is set to zero. A homogeneous Dirichlet boundary condition for the pressure is set at the outlet.

### 4.2. Bileaflet mechanical heart valve

#### 4.2.1. Geometry

**Figure 5:**
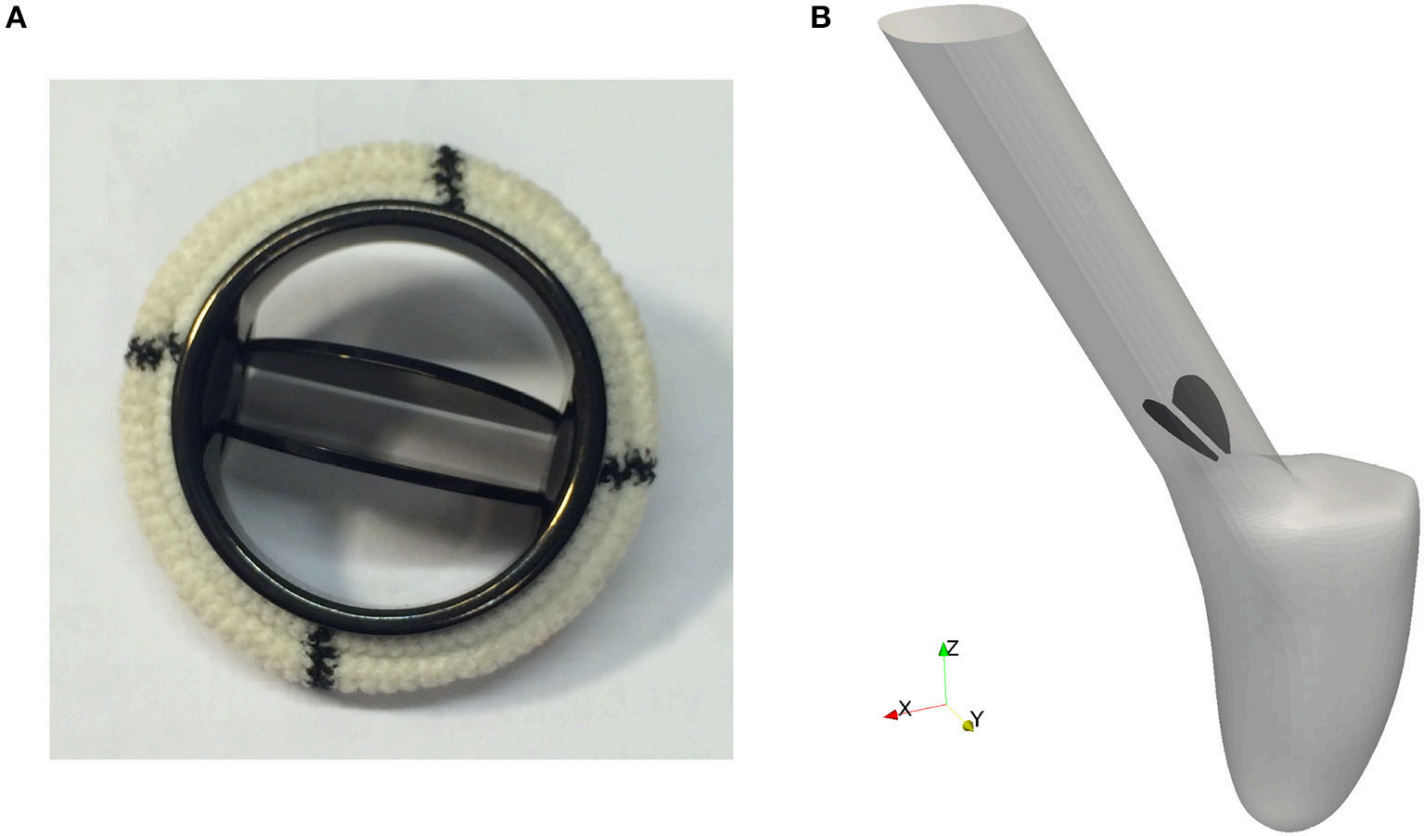
The geometry of a bileaflet mechanical heart valve. **(A)** Shows a typical bileaflet mechanical heart valve which is used as an artificial implant, and **(B)** is a simplified BMHV embedded in an idealized aorta used in our simulations.

Pathological conditions caused by valvular dysfunction in the form of a narrowing of the valve opening (stenosis) or insufficient closing of the leaflets, reduce the efficiency of the heart. To restore the hemodynamics function, the native heart valve may need to be repaired or even replaced by an artificial implant. Since the first clinical implantation of an artificial valve by Dr. Charles A. Hufnagel in 1952, many different mechanical and bioprosthetic valves have been developed. Due to their wear resistance, the bileaflet mechanical heart valves (BMHV) are most widely favored as aortic valve replacement. As can be seen in Figure 5A, a typical BMHV is made of a circular housing and two semicircular discs, which are mounted in the housing through a hinge mechanism. Both leaflets are rotating passively in response to the fluid dynamics resulting from the periodic contraction and expansion of the left ventricle.

Since feasibility, but not clinical validation is the focus of this paper, a detailed geometric model of a mechanical valve is secondary at this stage of investigation. Therefore, the BMHV models are reduced to the leaflets only, embedded in an idealized aorta as depicted in Figure 5B. The geometry is simplified in such a way that no contact between the leaflets occurs.

To use numerical simulations in order to study the flow through a mechanical prosthetic heart valve began in the early 1970s. Since then, many simulations of the flow dynamics around a BMHV have been conducted with the aim to elucidate and eliminate complications as thromboembolism. Simulating flow dynamics in the vicinity of a heart valve is a challenging task. The flow is pulsative and undergoes transition to turbulence. Patient-specific framework and the computational models should account for the multi-scale nature of the flow and deformability of the wall.

#### 4.2.2. Material

We apply the same material model as for the native valve and set the material parameters to $\mu_{s}=6.5\cdot 10^{5}MPa$ and ρ = 1, 000*kg*/*m*^3^.

#### 4.2.3. Initial and boundary conditions

Contrary to the native valve, we simulate the fluid-structure interaction of the leaflets and the hemodynamics of the left ventricle (LV) conjointly. A detailed description of the boundary conditions for the numerical simulation of the blood flow in the LV can be found in Spühler et al. (2015). We define a rotational axis by fixing two edge points of each leaflet. The hinges on which the leaflets are placed limit the rotational angle so that the BMHV is properly opened and closed. To mimic this mechanism, we set a threshold for the opening and closing angles respectively. As soon as a leaflet exceeds this angular barrier during systole or diastole, its position is locked. The leaflets are released from the fully open position if the mean pressure above the valve exceeds the mean pressure under the valve, and is disengaged from the closed position as soon as a new heart cycle starts. The maximal angular opening is set to 45°.

## 5. Results

**Figure 6:**
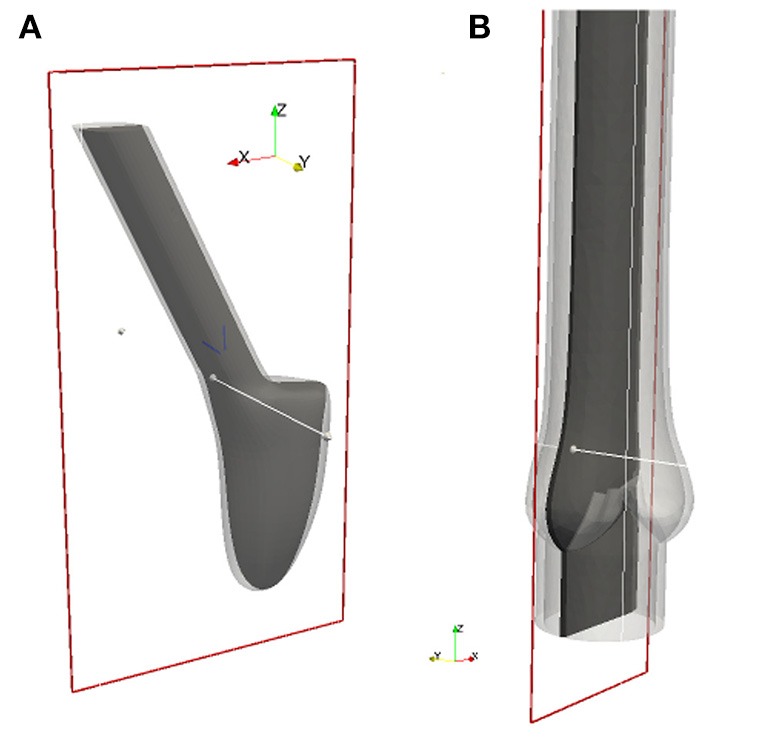
All 2-D images are visualized using these 2-D cuts in Paraview (Ahrens et al., 2005). The plane for the BMHV **(A)** is defined by its origin in (0.278, −1.65, 1.05) and normal (0, 1, 0), and the plane for the native valve **(B)** by its origin in (−0.179, 0.0, 1.05) and normal (0, 1, 0).

In this section we present the numerical results for the native and bileaflet mechanical valves. The 2-D cuts for the native valve and the BMHV are specified in Figure 6. Since we do not model the coronary arteries, which originate from the sinus of Valsalva, and the flow within the aortic root is almost quiescent during diastole, the results are based on the first heart cycle. The results for the native and mechanical valve are presented from meshes with 248′980 and 783′823′ vertices respectively. At the beginning of the simulation and during diastole, the time step size *k*^*n*^ is set such that the Courant-Friedrichs-Lewy (CFL) number is 0.5. During systole, we have to reduce *k*^*n*^ such that the mesh smoothing algorithms can maintain the mesh quality. No remeshing is required but in the worst case the CFL number had to be reduced to 0.01 to bypass a sensitive phase of large and fast deformation of the leaflets. To advance the solver one time step, the momentum and continuity equation, the Eikonal equation for contact detection, and the linear and non-linear elasticity equations for mesh smoothing have to be solved. When distributing ~2, 000 vertices per core, each sub-problem is solved in less than 0.5 s but its total time is about 5 s. The latter can slightly vary depending on the quality of the mesh since the cost of the non-linear elastic smoother is higher when the mesh quality is low.

### 5.1. Simulation results of the native valve

**Figure 7:**
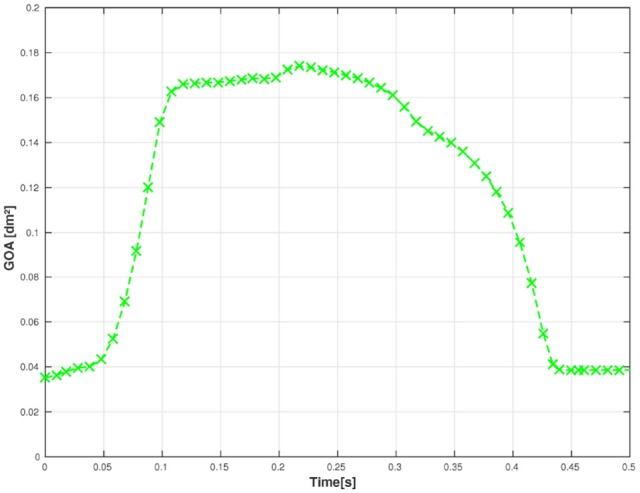
The geometric orifice area is plotted against time.

First, we examine the opening and closing movement of the aortic valve, which can be divided into four phases (Bellhouse and Talbot, 1969; Labrosse et al., 2010). A rapid valve opening time (RVOT) is followed by a period when the valve stays widely opened (quasi-steady phase). The valve first closes steadily and then rapidly due to reversed flow (RVCT) in the very end of systole. All these stages can be observed in our simulations by measuring the geometric orifice area (GOA), which is calculated by determining the area of the surface used for closing the valve. The time-dependent GOA is depicted in Figure 7 and matches well the dynamics captured in Labrosse et al. (2010). The rapid opening phase takes about 0.05 s and the valve stays open for about 0.15 s. Three-quarters of the valve closure is taking place when the flow is still flowing forward (~0.15 s) and a total closure is obtained by a small amount of reversed flow (~0.05 s).

**Figure 8:**
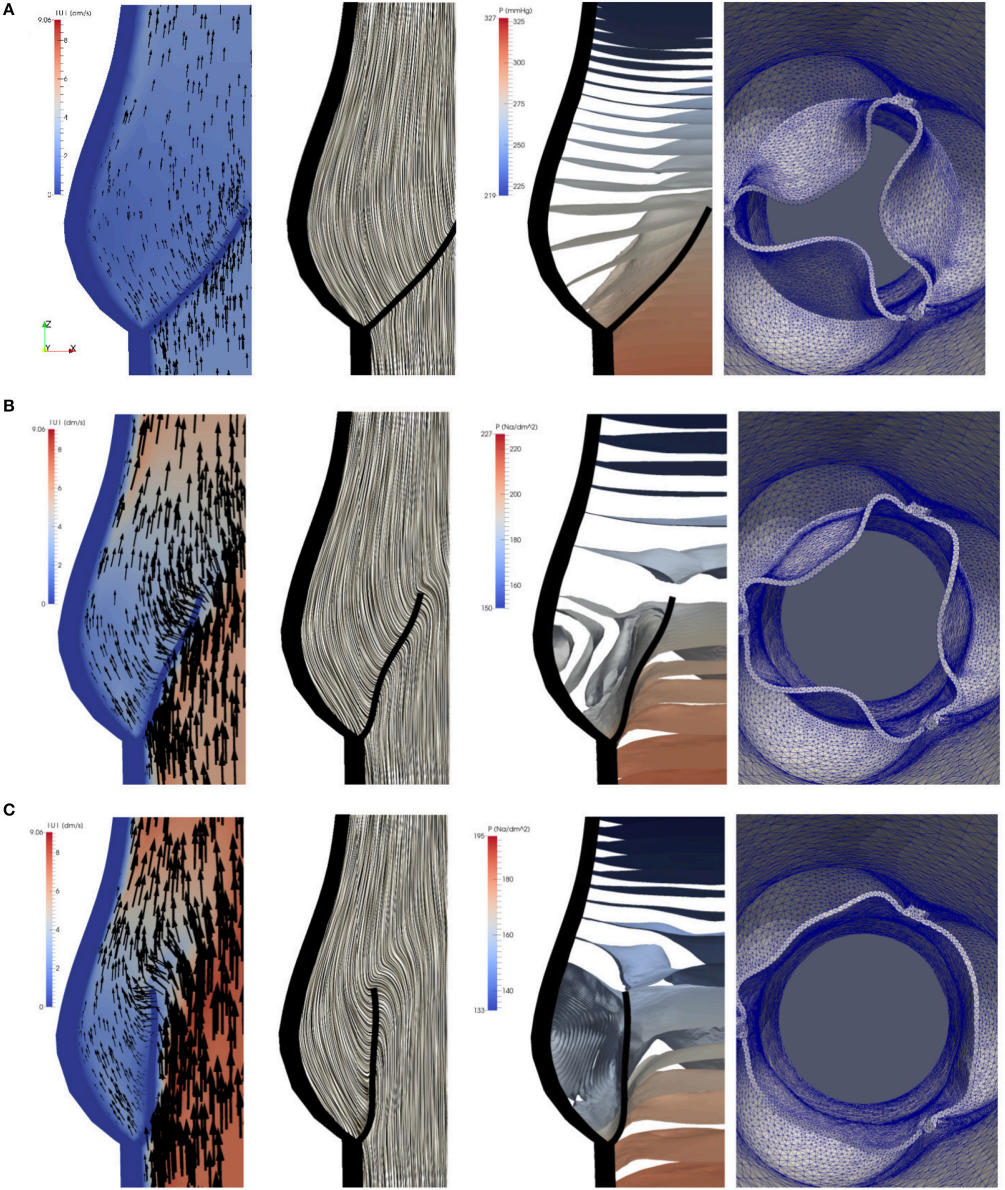
The instantaneous vector field of the velocity using arrows and line integral convolution (LIC), the pressure field and the aortic valve position during RVOT [*t* = 0.05 **(A)**, 0.08 **(B)**, 0.1 s **(C)**].

**Figure 9:**
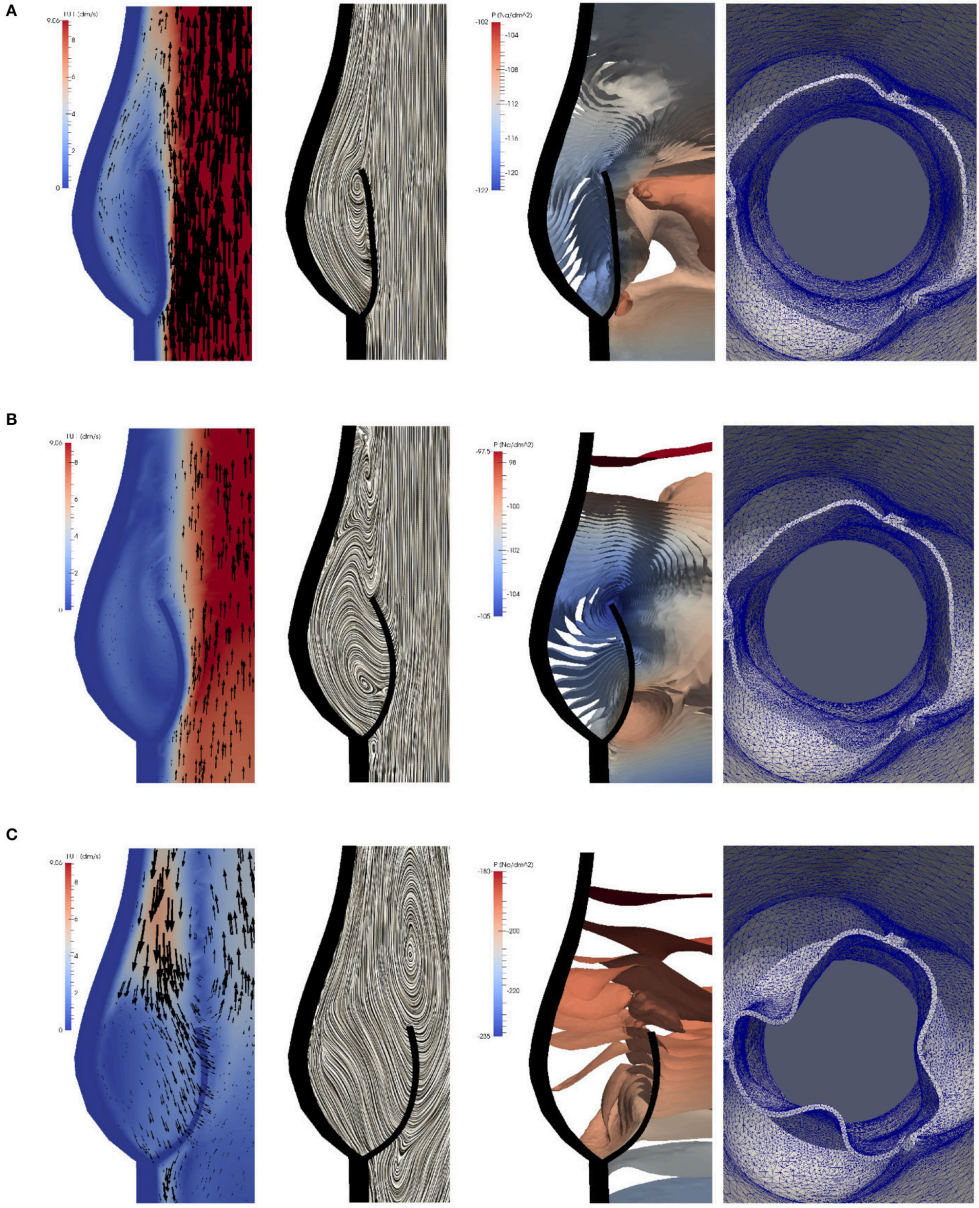
The instantaneous vector field of the velocity using arrows and line integral convolution (LIC), the pressure field and the aortic valve position in the phases of gradual closure [*t* = 0.25 **(A)**, 0.3 **(B)**] and RVCT [*t* = 0.4 s **(C)**].

To study the flow dynamics, in Figures 8, 9 the velocity and pressure fields together with the valve position are visualized at six time instances during the different phases: RVOT (*t* = 0.05, 0.08, 0.1 s), the phase of gradual closure (*t* = 0.25, 0.3 s) and RVCT (*t* = 0.4 s).

During RVOT, the fluid is accelerated over the whole domain flowing toward the outlet. As observed in De Hart et al. (2003), even the blood residing in the sinus cavity is washed out as shown in Figures 8A–C.

**Figure 10:**
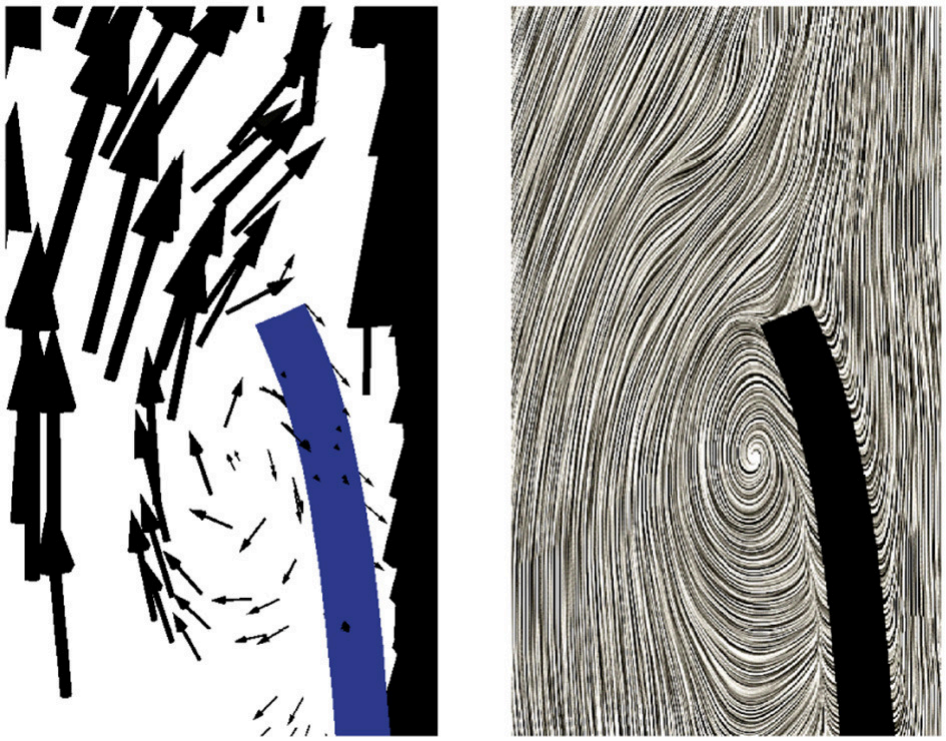
A small vortex is formed at the tip of the backside of the leaflet at *t* = 0.25 s as the flow starts to decelerate.

**Figure 11:**
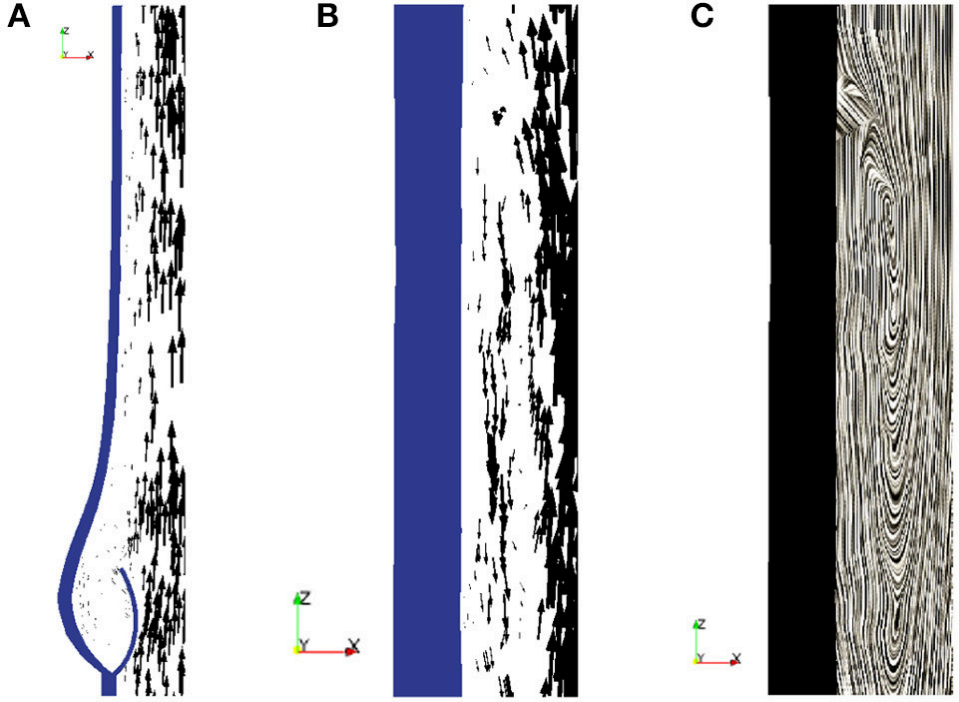
During the phase of deceleration a boundary between the outflowing jet **(A)** and regions with recirculating flow **(B,C)** can be observed. The figures show the velocity field at *t* = 0.3 s.

During the subsequent period, as the valve reaches and stays in the fully opened position, the flow is dominated by a strong, central jet. The flow starts to decelerate at about *t* = 0.2 *s* when the valve is still completely opened, and at about *t* = 0.25*s* the flow in the sinus cavity does not flow toward the outflow anymore, see Figure 9A. A small vortex starts to form at the tip of the backside of the leaflet, as depicted in Figure 10. Computing Lagrangian coherent structures, (Shadden et al., 2010) can distinguish two flow domains in this phase of deceleration. They observe a boundary between the strong outflowing jet and the regions with recirculating flow. These features can also be observed in our simulations as visualized in Figure 11.

**Figure 12:**
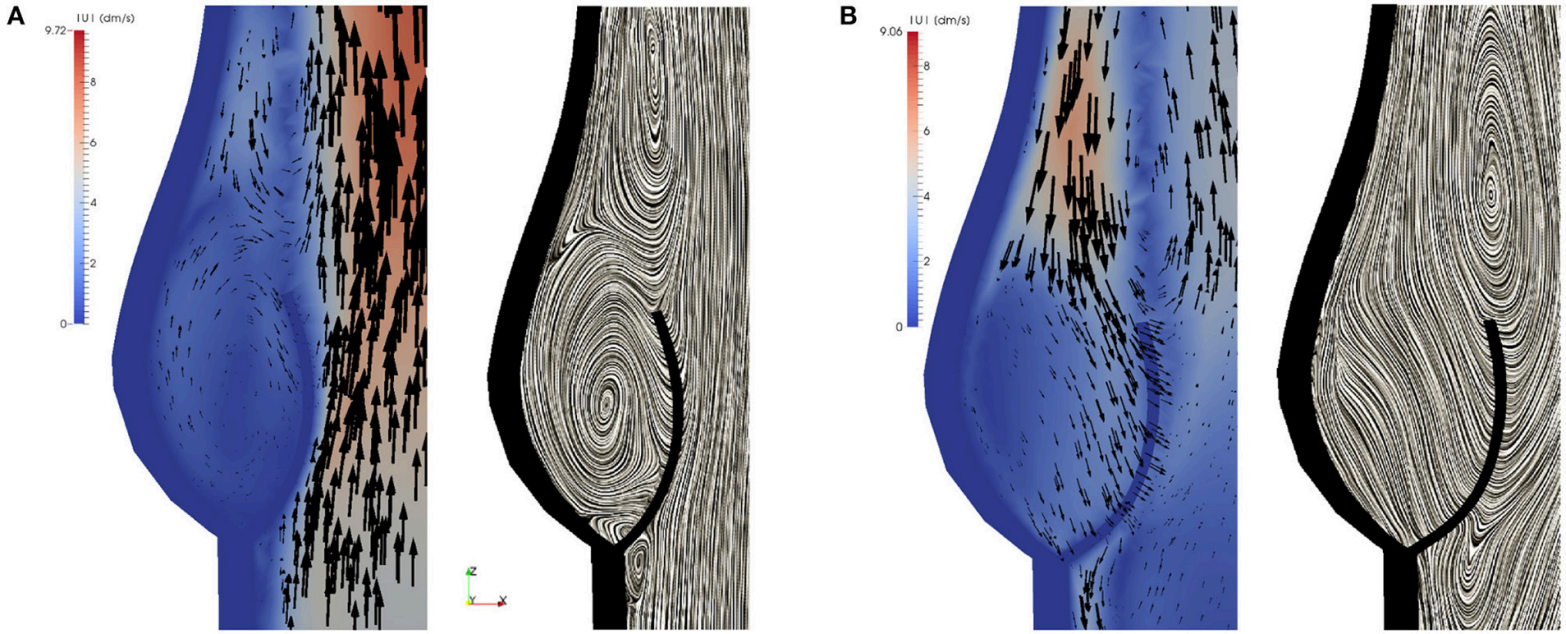
Two different vortices can be located during valve closure. Velocity field at *t* = 0.35 s **(A)** and *t* = 0.4 s **(B)**.

During the closing phase, two different vortices can be observed, as shown in Figures 9B,C, 12. One vortex is located just above the leaflet and the other one within the sinus cavity. Although they are rotating in counter directions, both of them drive the valve to close. The vortex within the sinus cavity merges to a streamline flow and only the vortex at the tip of the leaflet is left. A fully reversed flow in the ascending aorta is modeled by altering the inflow condition and a complete closure of the valve is achieved.

High stress has been connected to leaflet damage and failure. To analyze the stress distribution in the leaflets of our model, the von Mises stress τ_*v*_ in logarithmic scale is computed for the same time instances as the velocity and pressure fields in Figures 8, 9,

(16) $$\tau_{v}^{2}:\text{​}=\sum_{i,j=1}^{3}|\tau_{ij}-\delta_{ij}\frac{1}{3}tr(\tau ){|}^{2}.$$

**Figure 13:**
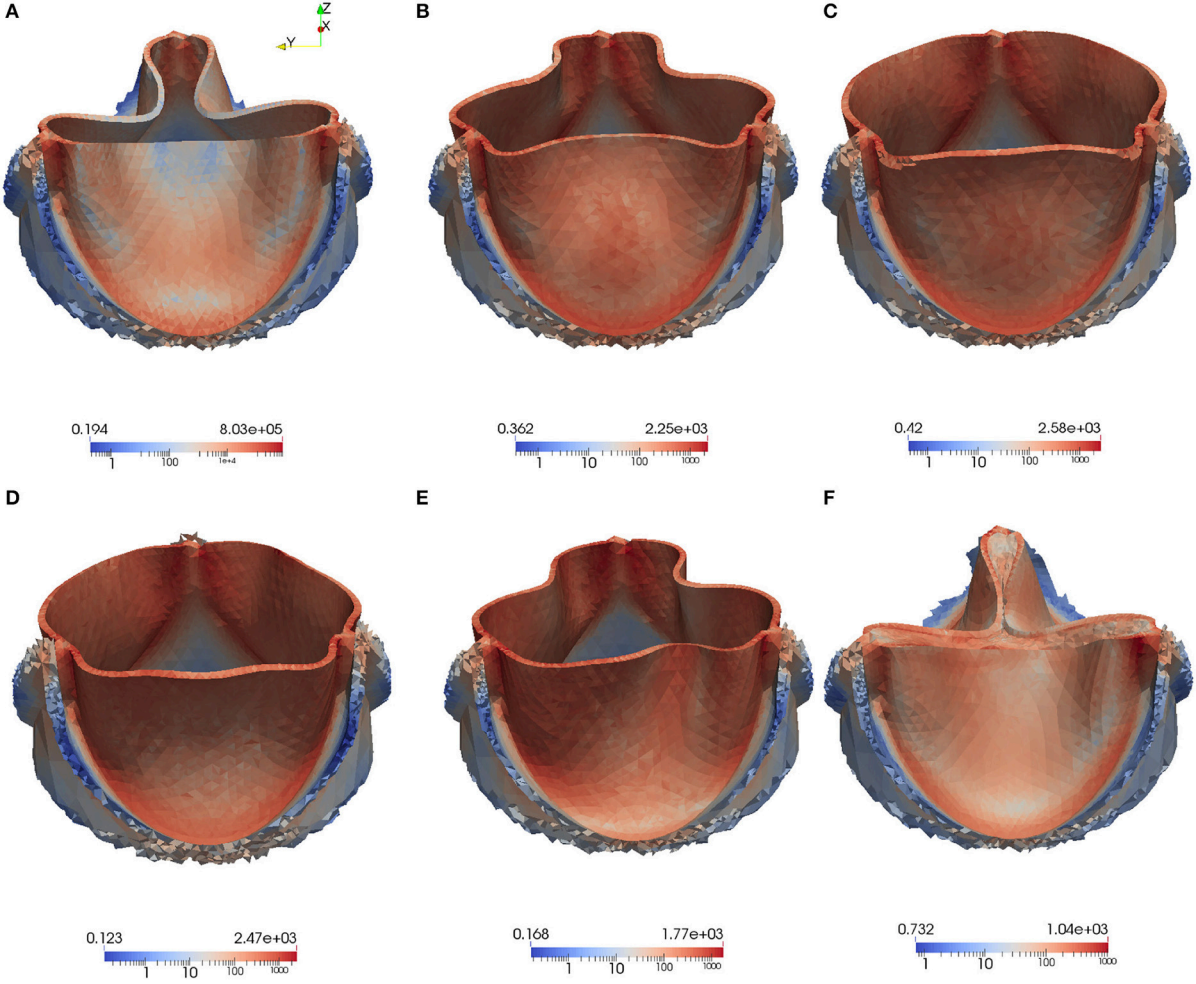
The von Mises Stress τ_*v*_ (Pa) is plotted at instantaneous time points during the acceleration phase, systole and deceleration phase: Leaflet position at *t* = 0.05 s **(A)**, *t* = 0.08 s **(B)**, *t* = 0.1 s **(C)**, *t* = 0.3 s **(D)**, *t* = 0.4 s **(E)**, *t* = 0.442s **(F)**.

We also visualize the stress distribution at the moment when the valve has just been closed at *t* = 0.442. As can be observed in Figure 13, regions with high stress can be localized to the attachment lines, commissure and leaflet belly. However, due to the low mesh resolution, this is only a qualitative analysis of the stress distribution.

No elaborated studies to analyze mesh sensitivity have been conducted yet. So far, we have only investigated to what extent the point of contact is affected by mesh refinement. For this purpose, the mesh is uniformly refined in the vicinity of the aortic root and we observe that the point of contact does slightly differ as listed in Table 2.

**Table 2:** Mesh sensitivity regarding the point of contact.

**Mesh**	**Mesh size**	**Point of contact in time (s)**
Mesh 1	37′631 vertices	0.440
Mesh 2	73′147 vertices	0.442
Mesh 3	248′980 vertices	0.434

### 5.2. Simulation results of the BMHV

**Figure 14:**
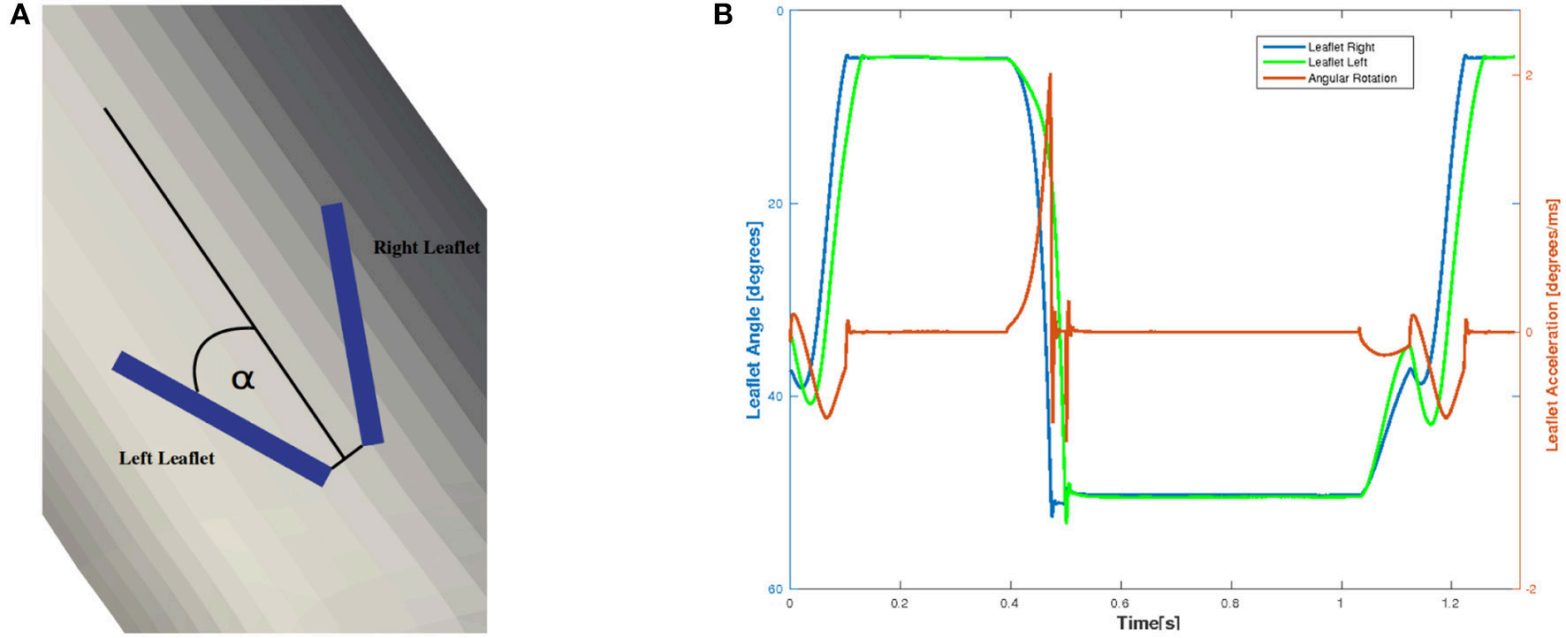
The definition of the rotational angle α is illustrated in **(A)** and the simulation results of the rotational angle and velocity are plotted in **(B)**.

To examine the valvular kinematics, we calculate the opening and closing angle as well as the rotational velocity of the leaflets. The rotational angle of the right and left leaflet is defined as depicted in Figure 14A, and the results are presented in Figure 14B. We observe that both leaflets are slightly open at first and accelerate and decelerate linearly while opening. The right leaflet precedes the left leaflet in the opening phase. This kinematic variation is of course strongly influenced by the geometry of the aorta. They then stay in their fully opened position until they close very rapidly, mainly due to backflow.

**Figure 15:**
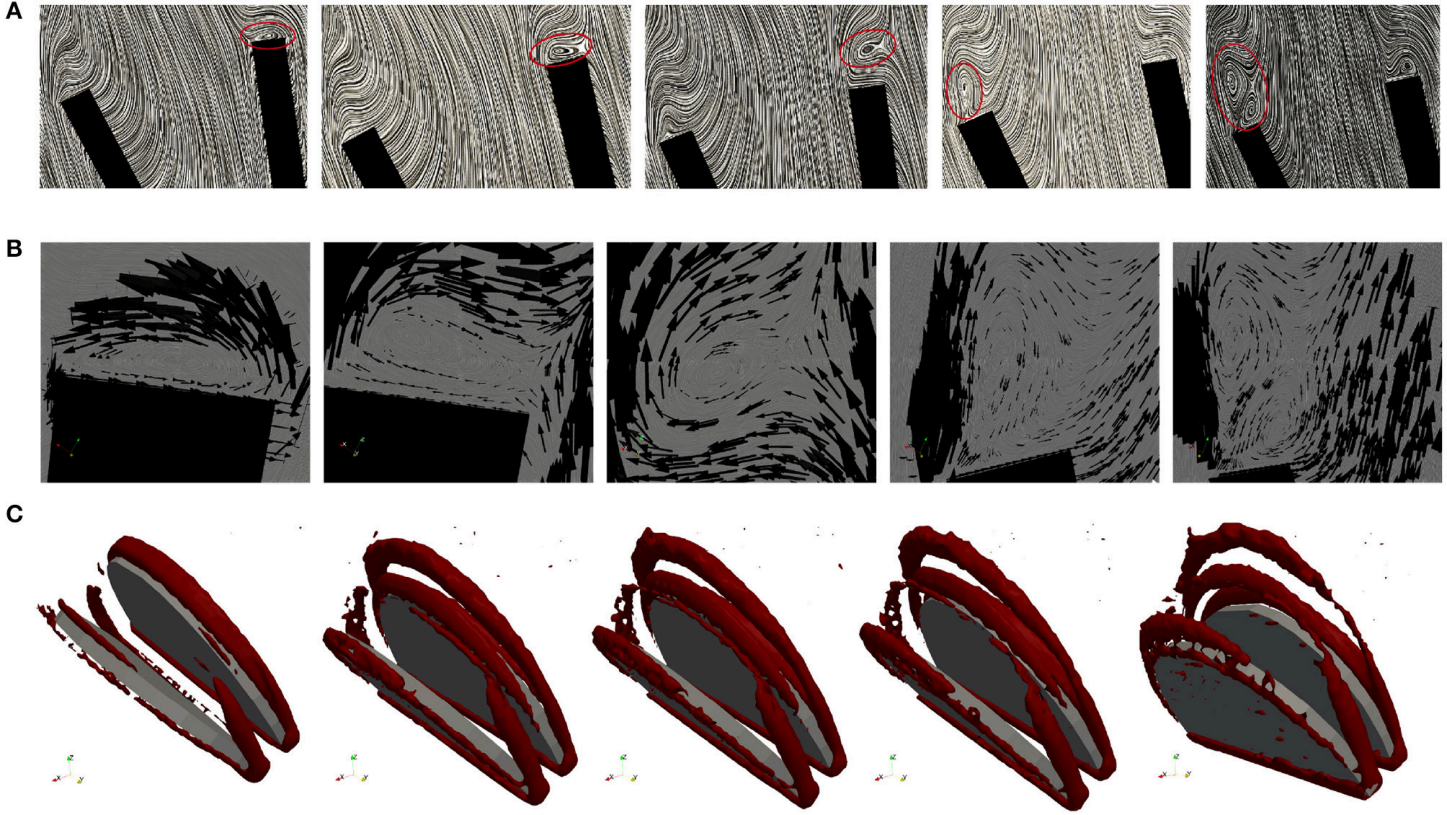
Snapshots of the velocity field and vortex structure of a BMHV at (from left to right) *t* = 0.1, 0.11, 0.115, 0.120, 0.124 s: Velocity field using LIC are depicted in **(A)**. The marked areas are enlarged and a close up view of circulations areas is shown in **(B)**. Three-dimensional vortex structures are visualized in **(C)** by using the λ_2_-criterion.

The geometry of a BMHV generates three jets, namely one central jet flowing through the gap between the leaflets and two side jets. During the end of the rapid opening phase, vortex rings are shed from the tip of the leaflets due to the difference in the velocity magnitude of the central jet and the two side jets. The vortex rings travel downstream a short distance before they vanish. Snapshots of the velocity field using line integral convolution (LIC) are visualized in Figure 15A. Figure 15B provides a closer view of the recirculation areas We use the open source code Saaz to calculate λ_2_ for our simulations (King et al., 2011). The threshold Θ_λ_2__ is manually adjusted until we can differentiate coherent vortex structures as shown in Figure 15C. The velocity vectors are added to indicate the rotational direction. The vortex observed at *t* = 0.1 at the right leaflet merges after a very short time into a recirculating flow with opposite direction (*t* = 0.11) and separates from the leaflet (*t* = 0.115). Meanwhile, a clockwise vortex is developed at the outer part of the left leaflet (*t* = 0.12), which eventually entails a neighboring, counter-clockwise rotating flow (*t* = 0.124). The former is swept off downstream, while the latter stays attached to the leaflet. When the valve has reached the fully opened position, no further vortices are developed.

## 6. Conclusion

The aim of our research is to develop an open source modular framework for modeling and simulating the blood flow in the heart. In the present work we place prototypes of a native and mechanical aortic valve between the left ventricle and the aorta.

We model both the fluid-structure interaction of the valve and the contact problem in the framework of a unified continuum. This approach to simulate the valvular dynamics is unique and has the advantageous properties that the whole problem can be described by a set of partial differential equations for which the same numerical methods are applicable. Furthermore, no instability issues due to the fluid-structure coupling is encountered. All algorithms are implemented in the FEniCS-HPC software framework optimized for parallel computing.

We generated a CAD model of an idealized native aortic root based on a small set of parameters, where we leave for future work to adapt the geometry to patient-specific data, and to connect the native aortic root to the left ventricle. The bileaflet mechanical heart valve is reduced to the leaflets only, which are embedded in a simplified geometric model of the aorta. In contrast to the native valve, we simulate the fluid-structure interaction of the leaflets and the hemodynamics of the left ventricle conjointly. The next step is a more realistic geometric model of the BMHV.

The weak point of our approach is the degradation of the mesh quality under large mesh deformations. All the simulations were conducted without remeshing, but we usually had to reduce the time step such that the mesh smoothing algorithms could comply with the deformation. The small time step size increased the computational time. Remeshing the volume mesh is an alternative, but not ideal for parallel computing. Thus, this limitation has to be addressed.

Although the material properties of both valves do not conform with realistic values yet, typical characteristics of the flow can be identified. Based on the simulation results, we conclude that our approach for simulating the fluid dynamics around aortic valves is feasible. More anatomically accurate models are targeted as a next step in order to not only examine the hemodynamics but also to test and optimize the design of valve implants. Simulations on larger meshes with higher resolution are to be performed to examine and strengthen the accuracy and robustness of our approach. Extension of the BMVH model, and connecting the native aortic root to a LV geometry, as well as simulations with much larger meshes, are aimed in our future work.

## Author contributions

JS had the main responsibility to implement and perform the simulations as well as to prepare the manuscript. The modeling was done in collaboration between JJ and JH, while NJ's work focused on parallel computing in FEniCS-HPC.

### Conflict of interest statement

The authors declare that the research was conducted in the absence of any commercial or financial relationships that could be construed as a potential conflict of interest.

The reviewer JA-S and handling Editor declared their shared affiliation.
